# Environmentally Friendly Strategies for Formulating Vegetable Oil-Based Nanoparticles for Anticancer Medicine

**DOI:** 10.3390/pharmaceutics15071908

**Published:** 2023-07-08

**Authors:** Nathália Freire, Raquel de Melo Barbosa, Fátima García-Villén, César Viseras, Luana Perioli, Rosana Fialho, Elaine Albuquerque

**Affiliations:** 1 Graduate Program in Industrial Engineering, Polytechnic School, Federal University of Bahia, Salvador 40210-630, Brazil; nathaliafreitasfreire@gmail.com (N.F.); rosanafialho@ufba.br (R.F.); elainecmca@ufba.br (E.A.); 2 Laboratory of Drug Development, Department of Pharmacy, Federal University of Rio Grande do Norte, Natal 59012-570, Brazil; 3 Department of Pharmacy and Pharmaceutical Technology, School of Pharmacy, University of Granada, Campus of Cartuja, 18071 Granada, Spain; fgarvillen@ugr.es (F.G.-V.); cviseras@ugr.es (C.V.); 4 Andalusian Institute of Earth Sciences, CSIC-University of Granada, Av. de las Palmeras 4, Armilla, 18100 Granada, Spain; 5 Department of Pharmaceutic Science, University of Perugia, 06123 Perugia, Italy; luana.perioli@unipg.it

**Keywords:** green chemistry, vegetable oils, bio-based nanoparticles, oncology APIs

## Abstract

The development of green synthesized polymeric nanoparticles with anticancer studies has been an emerging field in academia and the pharmaceutical and chemical industries. Vegetable oils are potential substitutes for petroleum derivatives, as they present a clean and environmentally friendly alternative and are available in abundance at relatively low prices. Biomass-derived chemicals can be converted into monomers with a unique structure, generating materials with new properties for the synthesis of sustainable monomers and polymers. The production of bio-based polymeric nanoparticles is a promising application of green chemistry for biomedical uses. There is an increasing demand for biocompatible and biodegradable materials for specific applications in the biomedical area, such as cancer therapy. This is encouraging scientists to work on research toward designing polymers with enhanced properties and clean processes, containing oncology active pharmaceutical ingredients (APIs). The nanoencapsulation of these APIs in bio-based polymeric nanoparticles can control the release of the substances, increase bioavailability, reduce problems of volatility and degradation, reduce side effects, and increase treatment efficiency. This review discusses the use of green chemistry for bio-based nanoparticle production and its application in anticancer medicine. The use of castor oil for the production of renewable monomers and polymers is proposed as an ideal candidate for such applications, as well as more suitable methods for the production of bio-based nanoparticles and some oncology APIs available for anticancer application.

## 1. Introduction

The polymer industry plays a significant role in our society as polymers have become essential materials nowadays. However, concerns over the extensive use of fossil-based raw materials, large amounts of reagents, and the accumulation of polymeric materials in the environment have increased. The need to release the polymer industry from its dependence on depleting resources is pushing the search for industrially applicable renewable alternatives [1].

Materials in the environment provide scientists and engineers with the possibility to change the polymerization process to develop a more sustainable society. Research has focused mainly on replacing fossil raw materials with renewable alternatives and developing end-of-life options that generate materials that are suitable for recycling or biodegradation [2].

One sustainable technology is the application of the principles of green chemistry to various processes. The design of chemical products and processes that reduce or eliminate the use and generation of hazardous substances is essential to living without having a negative impact on the environment. The sustainability evaluation of a product’s creation starts from the analysis of the feedstock used and its extraction. This highlights the importance of the seventh principle of green chemistry: “a raw material or feedstock should be renewable rather than depleting, wherever technically and economically practicable” [3].

A collaborative effort by industry, academia, and the government is needed to promote the adoption of the green chemistry technologies necessary to achieve a sustainable civilization. The progress of chemistry research, associated with the industrial revolution, has created a new scope for the preparation of novel polymeric materials based on renewable resources.

Biomass-derived chemicals can be converted into monomers with a unique structure, producing materials with novel properties, or modified in order to substitute commercial petroleum-based ones. Vegetable oils exhibit numerous reactive sites suitable for functionalization, including ester groups and double bonds present in unsaturated fatty acids, which can undergo chemical modifications through acrylation, transesterification, metathesis, and epoxidation reactions. These transformations enable the conversion of triglycerides into monomers capable of polymerization [4].

The most commonly encountered polymerization techniques for bio-based monomers are radical polymerization, condensation polymerization, and cationic polymerization. These techniques have been employed for the synthesis of diverse vegetable oil-based polymer types, including polyesters, polyamides, epoxies, and polyurethanes [5]. 

The fabrication of polymeric nanoparticles based on vegetable oils for biomedical applications can be achieved through various techniques. Among these, miniemulsion polymerization and solvent evaporation techniques are widely recognized and cited due to their inherent advantages, including process simplicity and stability.

Miniemulsion polymerization is a heterogeneous polymerization process used for the production of polymers in the form of nanoparticles for different applications of polymeric material. The thiol-ene reactions can be used in polymer and monomer synthesis and modification, side-chain/end-group modification, and preparation of various types of branched macromolecules. In the solvent evaporation technique, polymer solutions are prepared in a volatile solvent, and emulsions are formulated. These kinds of polymeric nanoparticles can be used in biomedical and pharmaceutical applications, such as antitumor therapy [1,6,7,8].

Nanoparticles have been of significant interest over the last decade as they offer great benefits for drug delivery to overcome limitations in conventional chemotherapy for anticancer treatments, for example. Nanoparticles for use as antitumor drug carriers have been in development due to their many advantages such as prolonging the biological circulation time, minimizing non-specific uptake, preventing undesirable side effects, improving cellular penetration, and allowing for specific cancer targeting [9].

A considerable amount of work has been conducted in search of novel cancer therapies using nanoparticle technology. Combined treatments employ either naturally active ingredients or drugs already intended for other uses so as to increase cell sensitivity to therapy and reduce drug toxicity, using a particular pharmaceutical combination and nanotechnology to develop drug delivery systems for targeting drugs to specific tumors [10]. 

This study aims to elucidate the application of green chemistry principles in the development of bio-based polymeric nanoparticles for anticancer therapy. Furthermore, it compiles the use of vegetable oils as sources of renewable monomers and polymers, highlighting castor oil as a promising candidate for such purposes. Additionally, it explores more suitable methodologies for the production of bio-based nanoparticles and discusses various oncology active pharmaceutical ingredients (APIs) with potential for anticancer applications.

## 2. Green Chemistry: Monomers and Polymers from Renewable Resources

The term green chemistry, as adopted by the IUPAC, is defined as the invention, design, and application of chemical products and processes to reduce or eliminate the use and generation of hazardous substances. Since their initial appearance in the scientific literature, the terms “green” and “sustainable” have been increasingly used and are nowadays present in several research areas. 

Green chemistry may be considered in the scientific and economical context in which academia, industry, and government are attempting to converge their efforts for the development of a sustainable civilization [11]. 

Green chemistry, also called sustainable chemistry, dates from 1991 when the U.S. Environmental Protection Agency (EPA) launched the Alternative Synthetic Pathways for Pollution Prevention research program under the auspices of the Pollution Prevention Act of 1990. However, the name green chemistry was officially adopted in 1996. 

American chemist Paul Anastas, one of the principal founders of green chemistry, claimed that by improving how chemicals are synthesized, it might be possible to prevent the production of pollutants. Together with John Warner in 1998, they created green chemistry’s 12 principles, including preventing waste wherever possible, designing chemicals that break down into harmless products after they are used, or using renewable feedstocks [12]. 

Fossil oil is consumed both in supplying energy as well as in the production of chemicals and polymers. Its extensive exploitation over the last 60 years has led to the cost-effective and easy creation of everyday products. 

The increase in world population and economic development, along with the decrease in the economically available amount of fossil oil, highlight the issue of its finite availability. With a regeneration time of several million years, fossil resources are extracted and consumed faster than they are produced and are thus considered non-renewable. Furthermore, environmental concerns related to their production and use, such as greenhouse gas emissions and the disposal of these non-degradable materials that led to serious environmental pollution, now motivate researchers to develop sustainable solutions [3,13].

The progress of chemistry research, associated with the industrial revolution, has created a new scope for the preparation of novel polymeric materials based on renewable resources, first through the chemical modification of natural polymers from the mid-nineteenth century, which gave rise to the first commercial thermoplastic materials, such as cellulose acetate and nitrate and the first elastomers, and second through the vulcanization of natural rubber. Later, these processes were complemented by approaches based on the controlled polymerization of a variety of natural monomers and oligomers [14].

The use of renewable raw materials, taking advantage of the synthetic potential of nature, can meet other principles of green chemistry, such as a built-in design for degradation or an expected lower toxicity of the resulting products [15]. Biomass-derived chemicals can be either converted into monomers with unique structures, leading to materials with novel properties, or modified in order to mimic commercial petroleum-based key molecules and monomers. Some of the most widely applied renewable raw materials in the chemical industry include plant oils, polysaccharides, sugars, wood, and others.

For instance, carbon dioxide is copolymerized with propylene oxide to generate propylene carbonate polyols. Terpenes, such as limonene, are chemically transformed to limonene oxide and copolymerized with carbon dioxide to generate poly(limonene carbonate). Triglycerides, from vegetable oils, are transformed into long-chain aliphatic polyesters. Natural carbohydrate polymers, such as starch, are broken down to glucose, which is subsequently transformed into polymers such as poly(ethylene furoate), polylactide, bio-derived poly(ethylene terephthalate), or bio-derived polyethylene. Products obtained from these renewables are as diverse as pharmaceuticals, coatings, packaging materials, or fine chemicals [2,3,15].

Vegetable oils represent one of the most interesting classes of renewables for the synthesis of sustainable monomers and polymers, as they are abundant and have relatively low prices, making them industrially attractive. Their long aliphatic chain contributes as a major element to the polymer backbone [1,3,15]. 

Biodegradable polymers are defined as polymers that are degraded and catabolized, eventually to carbon dioxide and water, by naturally occurring microorganisms such as bacteria, fungi, or algae. In addition, when they are degraded, these polymers should not generate any substances that are harmful to the natural environment.

Generally, natural materials or synthetic polymers that contain hydrolyzable bonds in the backbone, such as polyamides, polyesters, and polyether, are interesting candidates for biodegradation. 

Several parameters have been reported to influence the degradation behavior of biodegradable polymers, such as the chemical composition, molecular weight, and crystallinity of the polymer. Although the biodegradability of a material is independent of the origin of the starting raw materials used, biomass represents an abundant renewable resource for the production of biodegradable materials [13].

## 3. Synthesis of Monomers from Vegetable Oils

Vegetable oils are historically and currently the most important renewable feedstock of the chemical industry [16]. Due to their universal availability, inherent biodegradability, and low price, vegetable oils have become an area of intensive interest for both academic and industrial research as platform chemicals for polymeric materials [17].

**Figure 1:**
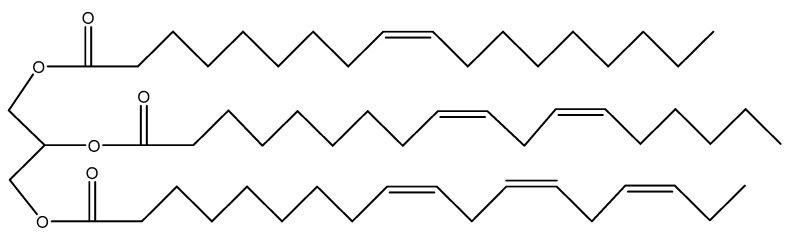
Structure of a polyunsaturated triglyceride.

The major components of vegetable oils are triglycerides (tri-esters of glycerol with long-chain fatty acids) with varying compositions of fatty acids depending on the plant, the crop, the season, and the growing conditions [15]. Vegetable triglycerides are among the most renewable resources exploited in science, in addition to other reasons, because of their unsaturated varieties [14]. The general molecular structure of triglycerides is demonstrated in Figure 1. 

Although triglycerides are found in almost all plants, the quantity that is available varies; for example, crops such as soybeans are estimated to yield only 20 wt% of triglycerides. Another challenge is that the chemical compositions of triglycerides vary both between and within a particular crop [2].

The physical and chemical properties of vegetable oils are mainly determined by the fatty acid chain length and the numbers and locations of double bonds in the fatty acid chains. The length of the fatty chain is usually between C12 and C20, with oleic acid (C18:1), linoleic acid (C18:2), and linolenic acid (C18:3) being the most common [17].

**Figure 2:**
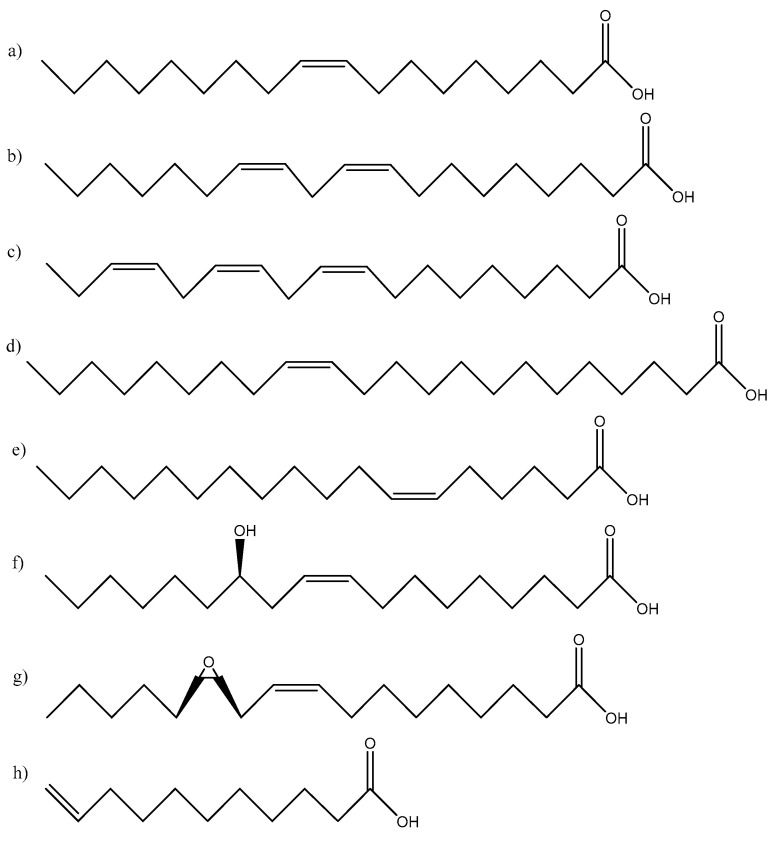
Fatty acids commonly used in polymer chemistry: (**a**) oleic acid, (**b**) linoleic acid, (**c**) linolenic acid, (**d**) erucic acid, (**e**) petroselinic acid, (**f**) ricinoleic acid, (**g**) vernolic acid, (**h**) 10-undecenoic acid.

The fatty acids account for 95% of the total weight of triglycerides, and their content is characteristic of each plant oil. The structures of some frequently studied fatty acids are depicted in Figure 2.

Fatty acids and esters can be easily obtained either by simple hydrolysis or alcoholysis of triglycerides. They are valuable renewable building blocks for the synthesis of designed monomers in the search for specific polymer properties that do not require extensive chemical modification prior to their application. 

There is a growing interest in the use of fatty acids as precursors of monomers, not only because of their renewability but also because of the properties they can provide to the final molecule [5].

The most common oil used in this kind of study is castor oil, due to the presence of hydroxyl group, and soybean oil, due to its low cost and high availability. Castor oil is a very versatile renewable feedstock for all kinds of polymeric materials, including polyesters, polyamides, polyurethanes, and many others. A process that has considerable potential is reacting to the alkene groups found in unsaturated fatty esters to produce α, ω-diene or α,ω-diols. Methyl 10-undecenoic acid, a castor oil-derived substance, was shown to be a suitable starting material for the preparation of esters with alkene groups that can produce biodegradable polymers [18].

## 4. Castor Oil as a Renewable Raw Material

Castor oil, from the castor plant (*Ricinus communis*), a native of tropical Asia and Africa, is one of the most exploited vegetable oils as a raw material for the chemical industry. It is naturalized and cultivated on a commercial scale all around the world in temperate zones. Like other plant oils, castor oil is extracted by a variety of processes or a combination of processes, such as different pressures and solvent extraction followed by a refining process.

The fatty acids of castor oil consist of up to 90% ricinoleic acid and varying small amounts of saturated and unsaturated fatty acids such as oleic acid, linoleic acid, and linolenic acid. 

The high content of ricinoleic acid is the reason for the high value of castor oil and its versatile application possibilities in the chemical industry. From castor oil processing, like from other applications of vegetable oils, glycerol is obtained as a byproduct, which is a platform chemical with widespread application possibilities in cosmetics, pharmaceuticals, detergents, the production of resins and additives, and the food industry [19]. For instance, certain characteristics of castor oil, namely high lubricity, high viscosity over a wide range of temperatures, and insolubility in aliphatic petrochemical fuels and solvents, make it directly applicable as a lubricant, coating, ink, polymer, and foam. 

Biotechnology offers ways to alter the composition of castor oil fatty acids for processes in the chemical industry with an emphasis on development and application in polymer science. There are several possible chemical transformations of castor oil depending on the reacting functional group. Ester reactions include hydrolysis, esterification, alcoholysis, saponification, reduction, amidation, and halogenation; double bond reactions include oxidation, polymerization, hydrogenation, epoxidation, halogenation, addition reactions, sulfonation, and metathesis; and hydroxyl group reactions include dehydration, hydrolysis, caustic fusion, pyrolysis, alkoxylation, esterification, halogenation, urethane formation, and sulfonation [20].

**Figure 3:**
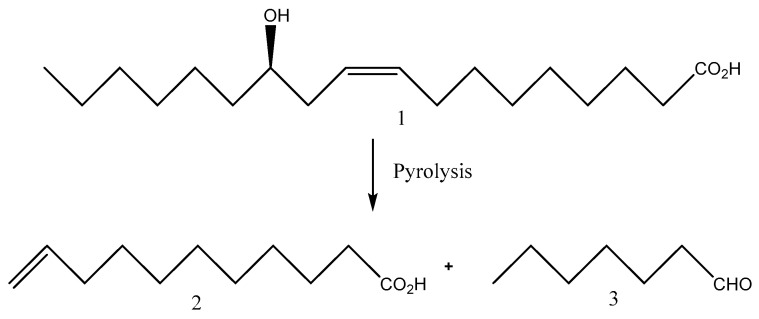
Products of the thermal fragmentation of ricinoleic acid. (1) Ricinoleic acid, (2) 10-undecenoic acid, (3) heptanal.

The pyrolysis of ricinoleic acid at high temperatures (>350 °C) splits the ricinoleate molecule at the hydroxyl group to form heptaldehyde and undecenoic acid (Figure 3), which is a platform chemical that can be used to synthesize a large variety of renewable monomers and polymers [20,21,22].

The use of castor oils as a raw material in the synthesis of polymeric materials is very well-established. Castor oil polymers are applied in various fields such as wound dressing, drug delivery, bone tissue engineering, and membranes for fuel cell fabrication [23].

A vast array of copolymers is viable when castor oil (or ricinoleic acid) is combined with other monomers. Materials with varied properties can be obtained by tweaking the chemistry of these copolymers. Altering of comonomer compositions leads to polyesters with controlled mechanical, thermal, and viscoelastic properties, as well as degradation profiles [24]. 

Ref. [25] synthesized a bio-based monomer acrylate ricinoleic acid from castor oil and copolymerized it with methyl methacrylate in miniemulsion, forming polymeric nanoparticles. The addition of the bio-based monomer led to a decrease in the glass transition temperature of the copolymer and to the formation of a small fraction of gel, resulting in materials with interesting properties for future applications, such as pressure-sensitive adhesives.

In the medical field, biodegradable aliphatic polyesters are the preferred materials as biomaterials because of their biodegradation and biocompatibility. Ref. [26] obtained biocompatible polymeric nanoparticles via thiol-ene polymerization in miniemulsion using a fully renewable α,ω-diene monomer obtained from 10-undecenoic acid and 1,3-propanediol; both were derived from castor oil. 

Additionally, in the biomedical application of polymers nanoparticles, Ref. [27] synthesized poly(thioether-ester) nanoparticles via thiol-ene miniemulsion polymerization using a bio-based α,ω-diene monomer, namely dianhydro-d-glucityl diundec-10-enoate, synthesized from 10-undecenoic acid (derived from castor oil) and isosorbide (derived from starch). These kinds of polymer nanoparticles have tremendous scope for further fabrication for the biomedical application area, including studies for anticancer treatments.

## 5. Polymeric Nanoparticles and Some Production Techniques

Nanoparticles are frequently defined as solid, colloidal particles in the range of 10–1000 nm. This is a collective term given for any type of polymer nanoparticle, but specifically for nanospheres and nanocapsules. 

Nanocapsules act as drug reservoirs due to their vesicular structure, in which the retained active pharmaceutical ingredients are reserved in an aqueous or non-aqueous liquid core placed in the vesicle cavity and enclosed by the solidified polymeric shell. While nanospheres are matrix particles, particles whose entire mass is solid and molecules may be adsorbed at the sphere surface or encapsulated within the particle [8,28].

The field of polymer nanoparticles assumes a significant role across a broad spectrum of disciplines, encompassing electronics [29], conducting materials [30], medicine [31,32], and biotechnology [33,34].

Polymers are very convenient materials for the production of nanoparticles with many potential medical applications. The polymers used in the preparation of nanoparticles should be compatible with the body in terms of adaptability and biodegradability. The most commonly used natural polymers in the preparation of polymeric nanoparticles are chitosan, gelatin, sodium alginate, and albumin. Synthetic polymers are mostly represented by polylactides (PLAs), polyglycolides (PGAs), poly (lactide co-glycolides) (PLGAs), polyanhydrides, polyorthoesters, polycyanoacrylates, polycaprolactone, poly glutamic acid, poly malic acid, poly (N-vinyl pyrrolidone), poly (methyl methacrylate), poly (vinyl alcohol), poly (acrylic acid), poly acrylamide, poly (ethylene glycol), and poly (methacrylic acid). Although there are many possible polymers, the application of the derivatives of castor oil, such as 10-undecenoic acid, for the preparation of monomers used in the production of polymer nanoparticles has increased [28,34].

Polymer nanoparticles can be conveniently prepared either from preformed polymers or the direct polymerization of monomers using classical mechanisms. Methods such as solvent evaporation [35], salting-out [36], dialysis [37], and supercritical fluid technology [38] can be utilized for the preparation of polymer nanoparticles from preformed polymers. 

**Figure 4:**
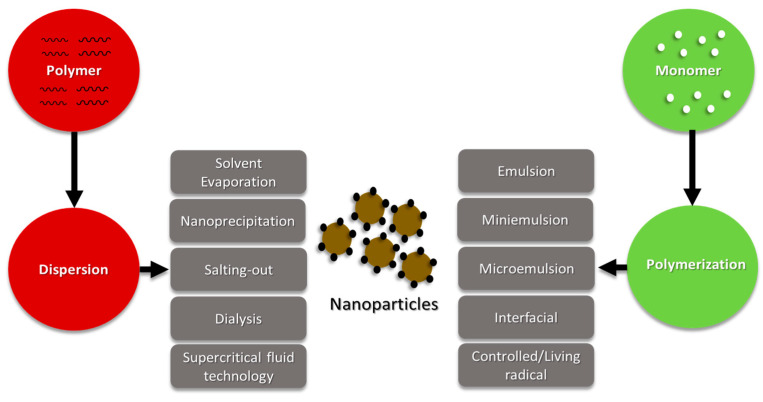
Schematic representation of various techniques for the preparation of polymer nanoparticles.

On the other hand, polymer nanoparticles can be directly synthesized by the polymerization of monomers using various polymerization techniques such as microemulsion, miniemulsion, and interfacial polymerization (Figure 4) [8]. 

### 5.1. Solvent Evaporation Technique 

**Figure 5:**
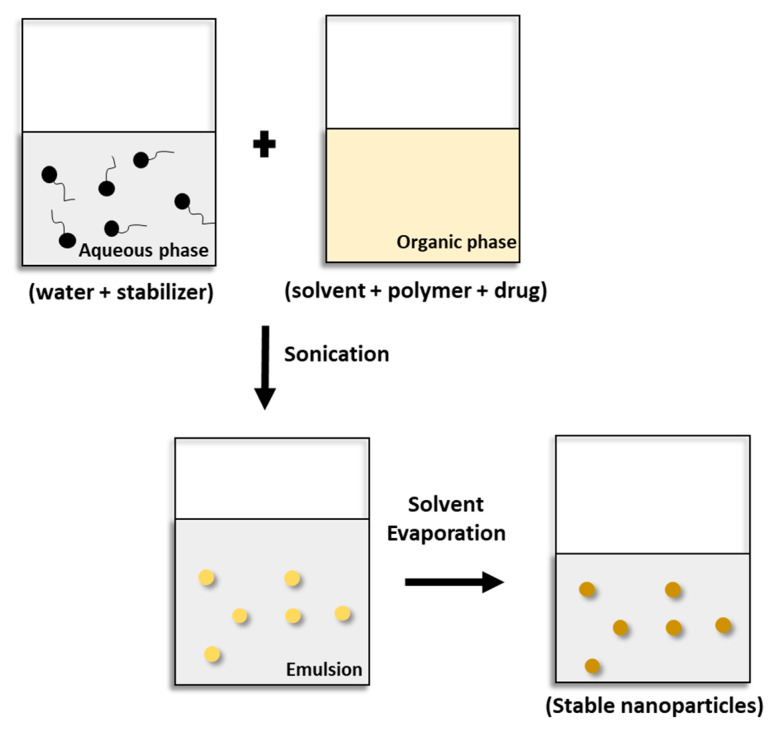
Scheme of the emulsification solvent evaporation technique.

The emulsification solvent evaporation technique was first reported in 1981 [39]. Hydrophobic polymers (synthetic, semi-synthetic, or natural) and drugs (usually lipophilic) are dissolved in an organic solvent (e.g., chloroform, dichloromethane, ethyl acetate), which is volatile and water-immiscible. This solution is then emulsified in an aqueous stabilizer solution. Emulsification is carried out by sonication or under high-energy homogenization to reduce the size of the emulsion droplets, and an emulsion is formed. The organic solvent is then removed by evaporation at room temperature under stirring or under reduced pressure. Afterward, the solidified nanoparticles can be collected by ultracentrifugation and washed with distilled water to remove additives, such as surfactants (Figure 5) [8,39,40,41,42].

Solvent evaporation is the most commonly used technique for the preparation of the nanoparticles of polymers in the current literature on techniques using a dispersion of preformed polymers [43,44,45]. In the polymerization of monomers, the number of publications on miniemulsion polymerization and the development of a wide range of renewable polymer materials has recently increased substantially [8].

### 5.2. Miniemulsion Polymerization

Miniemulsion is part of the emulsified polymerization systems, and its main characteristic is the size of the drops and the stability of the final emulsion. A nanoemulsion can be considered a conventional emulsion containing very small particles (size ranging from 50 to 500 nm) [46,47].

Ref. [48] were pioneers in the study of polymerizations in miniemulsions, describing the polymerization process in monomer drops. Their discussions led to speculation about the possibility of nucleation and polymerization in very small monomer droplets during emulsion polymerization.

Asua (2002) defined miniemulsions as dispersions of small monomer drops in water, stabilized by a surfactant against the coalescence of the drops by the action of the Brownian motion (a union of two or more drops occurring the rupture of the interface and resulting in a larger drop) and a co-stabilizer to minimize diffusional degradation (Ostwald Ripening, a process in which small drops are grouped by the difference of pressure, leads to an increase in the average size of droplets) [49]. 

A typical formulation includes water, a monomer, co-stabilizing (when used), a surfactant, and an initiator (which can be soluble in the aqueous or organic phase). The surfactant is dissolved in water, the active to be encapsulated is dissolved in the monomer, and both are mixed under agitation. A shear mechanism (homogenization) is required to ensure the submicrometric size of the drops [46].

The mechanical homogenization of miniemulsions can be obtained by different methods. Initially, simple agitation was used as the main means of homogenization. Subsequently, the use of omni-mixers and ultra-turrax was cataloged. However, the energy transferred by these techniques is not enough to obtain small drops distributed homogeneously. Much higher energy for the fragmentation of large drops into small ones is required. Currently, ultrasonication is used, especially for the homogenization of small quantities, while micro-corrugators or high-pressure homogenizers are favorable for large quantities of emulsion [46].

**Figure 6:**
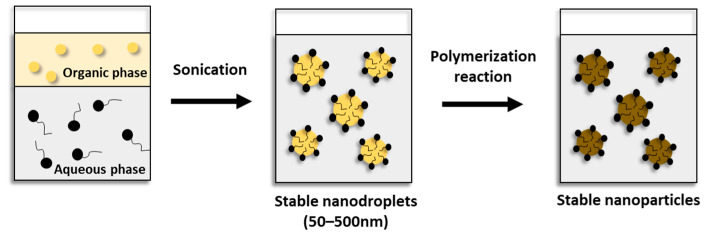
Scheme of the miniemulsion process. Source: adapted from [51].

In the first stage of the miniemulsion polymerization process, small drops are formed by a system containing the dispersed phase (a monomer, active to be encapsulated, and a co-stabilizer) and continuous phase (aqueous phase with a surfactant). The initiator can be added in the dispersed phase or continuous phase, depending on whether it is hydro- or organic-soluble. The surface area of the droplets in these systems is very large, and most surfactant is adsorbed on the surface of the droplets [50]. In the second step, the drops are nucleated and polymerized [51,52]. In Figure 6, the scheme of the miniemulsion polymerization process is demonstrated.

## 6. Thiol-Ene Polymerization for Nanoparticle Production

Thiol chemistry, a versatile tool, was first described in 1905 by Posner. The author reports the thiol coupling to different types of mono- and bi-unsaturated compounds such as aliphatics, aromatics, terpenes, and hydroaromatics. The thiol-ene free radical addition is of special interest due to its application range and simplicity. Early work in this field appeared in the late-1930s to early-1950s [53].

A patent concerning the polymerization of dithiols and dialkenes via radical additions dates back to 1941. The reaction is well-known to proceed via a free-radical mechanism. Generally, radical reactions are known to be quite fast reactions, and thiol-ene additions offer some additional features, such as robustness and efficiency, which has meant that this reaction is considered one of the click reactions, and it has become very popular in recent years [54].

**Figure 7:**
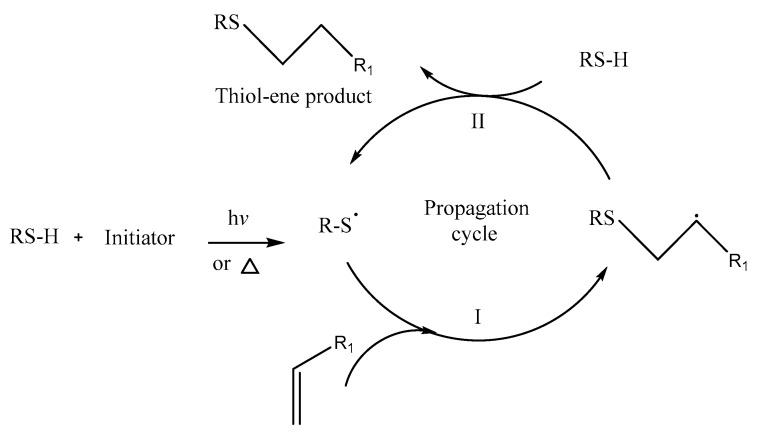
The mechanism for the hydrothiolation of a C=C bond in the presence of an initiator.

Like a traditional free-radical polymerization, thiol-ene polymerization reaction proceeds in three stages: initiation, propagation, and termination, plus a chain transfer step. At initiation, the formation of thiol groups occurs by removing hydrogen. During propagation, the thiol radical is added to the unsaturated moiety (ene) group of the olefin, which generates an unpaired electron in the central carbon of the chain. Chain transfer occurs when the central carbon donates the electron to the thiol group, producing another thiol group, thereby restoring the mechanism (Figure 7). Termination occurs through radical-radical coupling [55].

The efficiency of this reaction, therefore, requires unsaturation in the terminal position and strongly depends on the thiol compound used. As mentioned above, the propagation step of this reaction is the addition of a thiyl radical to a C=C double bond and the subsequent abstraction of a hydrogen atom by the formed carbon radical from another thiol compound, forming a new thiyl radical. The formation of the carbon radical is reversible and a rate-determining step, which explains the low reactivity of internal alkenes [54]. 

There has been impressive growth in the use of the thiol-ene reaction in polymer synthesis and modification. Examples include use in monomer synthesis and side-chain/end-group modification, the preparation of various types of branched macromolecules, the preparation of inorganic–organic composites, nanoparticle modification, surface modification, bio-related applications, and cross-linked polymers [6].

Cases of reactions between vegetable oils or derivatives and thiols found in the scientific literature [18] describe the use of methyl-10-undecenoate, a castor oil derived in thiol-ene reactions. A variety of renewable monomers were obtained in high yields. Their polymerization was also studied, and the material properties of the resulting polyesters were investigated, revealing good thermal properties, making them possible candidates for the substitution of petroleum-based materials. Ref. [56] developed a methodology that was applied to a biomass-derived monomer of 10-undecenoic acid. Thiol-ene click step growth polymerization was used to prepare alkene-functionalized linear polymers with variable molar mass.

Ref. [57] developed multi-responsive cross-linked core poly(thioether ester) micelles. Firstly, a poly (thioether ester) was synthesized by the thiol-ene polymerization using ethanedithiol and glycidyl methacrylate as monomers. The resultant poly (thioether ester) was then coupled with carboxyl terminated poly (ethylene glycol) (PEG) and lipoic acid to give a graft a copolymer that could self-assemble into micelles in the aqueous media and turn into cross-linked core nanoparticles in the presence of dithiothreitol. The cross-linked core micelles showed a more compact structure and higher drug-loading efficiency compared with non-cross-linked micelles. These results indicate that the cross-linked micelles may have considerable potential for controlled drug delivery in cancer therapy.

In [58], cationic polymeric nanocapsules were generated as potentially therapeutic nanocarriers. These nanocapsules were synthesized from allyl-functionalized cationic polylactide (CPLA) by efficient UV-induced thiol-ene interfacial cross-linking in transparent miniemulsions. These nanocapsules can effectively bypass the multidrug resistance of cancer cells, thereby resulting in increased intracellular drug concentration and reduced cell viability.

In virtue of some of the aforementioned advantages of thiol-ene reactions, such as that they can be carried out under mild conditions, the possibility of producing cross-linked and functionalized structures and improving degradability, these kinds of reactions are considered environmentally friendly candidates for synthesizing biocompatible and biodegradable polymers for biomedical application, such as cancer therapy [26,59,60,61].

The use of in situ miniemulsion polymerization (polymerization of a monomer and encapsulation of activity at the same time) by thiol-ene has been investigated. Nanoparticles have several applications: pharmaceutical, biomedical, and cosmetic. The development of polymeric nanoparticle formulations containing anticancer-like actives, for example, is relevant here. This type of system has the potential to enhance the bioavailability of encapsulated substances and mitigate issues associated with premature degradation. Furthermore, the surface functionalization of nanoparticles, such as protein conjugation, can be employed to promote prolonged circulation in the biological milieu and facilitate targeted delivery to specific sites.

## 7. Application of Polymeric Nanoparticles in Cancer Therapy

Nanoparticles have been of significant interest over the last decade, as they offer opportunities for new drug delivery systems. Recently, nanoparticles have been extensively employed as biomaterials because of their favorable characteristics in terms of simple elaboration and design, good biocompatibility, and a broad structure variety [9]. Nanoparticles can be considered ideal candidates for cancer therapy in comparison with other possibilities, such as chemotherapy [28].

Chemotherapy is a predominant cancer treatment strategy in which anticancer drugs are used to induce cell death in cancer cells. However, it has several limitations, such as requiring a high drug dose, causing adverse effects, and multidrug resistance, which can reduce the efficacy of the therapy. To overcome the limitations associated with chemotherapy, nanomedicine strategies employing the formulations of anticancer drugs in various nanocarrier forms have been reported [62].

The first clinical trial of nanoparticles for anticancer drug delivery took place in the mid-1980s, and the first nanoparticles (e.g., liposomal with encapsulated doxorubicin) entered the pharmaceutical market in 1995. Since then, numerous new nanoparticles for cancer drug delivery have been under development given their many advantages, such as enhancing the solubility of hydrophobic drugs, prolonging circulation time, preventing side effects, improving intracellular penetration, and allowing for specific cancer targeting [9].

In Table 1, recent uses of nanoparticles for cancer therapy are listed. Polyhydroxyalkanoates (PHAs) are natural, non-toxic, biodegradable, and biocompatible polyesters. Cyclodextrin (CD) and its derivatives are natural cyclic oligosaccharides, and poly(lactic-co-glycolic acid) (PLGA) is a copolymer of lactic acid and glycolic acid.

The use of nanoparticles in conventional chemotherapy is recognized and has been accepted by the FDA (Food and Drug Administration) for broader usage. Anticancer drug entrapment within nanoparticles guards them against efflux transporters, and the nano-sized range accelerates their entrance through biological membranes. Additionally, the polymer shell protects the drug against the body’s enzymes. Current developments in nanotechnology have revealed many types of targeting strategies for augmenting drug accumulation in the tumor while restricting the undesirable toxicity to normal cells. As the nanoparticles are designed for targeted drug delivery systems, they increase the anticancer active ingredients delivered to tumors without affecting non-cancerous regions [28].

Some of the applications of nanoparticles in cancer therapy can be seen in the work of [62], who developed a novel biodegradable antibody-conjugated polymeric nanoparticles designed for targeted delivery in breast cancer receptors. The formulated nanoparticles were capable of sustained pH-dependent drug release. The results indicated that the formulated nanoparticles were found to provide better anticancer and inhibitory activity against breast cancer cells than the free anticancer agent by in vitro and in vivo evaluations. 

Ref. [125] evaluated the inhibition of glioma growth in vivo by combining interstitial chemotherapy and the targeting drug delivery strategy. They developed 3-bis(2- chloroethyl)-1-nitrosourea-loaded wafers that were implanted in the tumor and 3-bis(2- chloroethyl)-1-nitrosourea-loaded poly(lactic acid) nanoparticles decorated with transferrin that were administrated by intracarotid perfusion. The results showed that the combined therapy significantly prolonged the survival time of glioma-bearing rats in comparison with either treatment alone.

Ref. [126] synthesized and characterized zinc (II) phthalocyanine loaded poly(methyl methacrylate) obtained by miniemulsion polymerization for photodynamic therapy in leukemic cells. The cytotoxicity and phototoxicity studies indicated that the nanoparticles improved the photobiological activity of zinc phthalocyanine on leukemic cells. Although good results of zinc (II) phthalocyanine loaded poly(methyl methacrylate) were obtained for photodynamic therapy, the poly(methyl methacrylate) is not a biodegradable polymer. This boosted other works with new kinds of renewable and biodegradable polymer, such as poly(thioether-ester).

For these reasons, nanoparticles can be used in many applications in cancer remediation. There are a multitude of possibilities for nanoparticle technology that need to be explored to harness their remarkable use in applications as a new class of targeted remediation for cancer therapy [127,128,129,130,131].

Nanoparticles containing oncology APIs offer a different alternative to conventional treatments, mostly due to their targeted delivery and action. They can also be used as biosensors, enabling cancer detection or carriers in targeted drug delivery to specific locations [132].

Oncology or anticancer APIs, also called antineoplastic drugs, refer to the biologically active components present in anticancer drugs. They are effective in the treatment of malignant or cancerous diseases. There are several major classes of oncology APIs. These include alkylating agents, antimetabolites, natural products, and hormones, which demonstrate anticancer activity and are used in the treatment of malignant diseases.

The nanoencapsulation of oncology APIs exhibits other advantages over conventional medical methodologies. For example, they enter selective tissue at the molecular level, provide a large surface area and high absorption rate, increase cellular uptake and drug localization, provide accurate and targeted drug delivery to cancerous cells without interactions with healthy cells, use a lower dosage due to the encapsulation of drugs or small molecules, improve the uptake of poorly soluble drugs, decrease medicinal toxicity, and minimize or suppress the resistance arising from the physiological barriers in the body [132,133].

For these and other reasons already mentioned, new oncology APIs have been studied in cancer treatment strategies. Table 2 lists examples of new oncology APIs for cancer treatment. These APIs possess significant potential to enhance cancer treatments, and the nanoencapsulation of these agents can optimize their anticancer efficacy.

## 8. Conclusions

The need to release the polymer industry from its dependence on non-renewable resources is a significant concern. Vegetable oils represent one of the most interesting classes of renewables for the synthesis of sustainable monomers and polymers that can be applied for biomedical use as nanoparticles containing active pharmaceutical ingredients for anticancer therapy. Nanoparticles are rapidly changing the direction of cancer treatment; they can deliver oncology APIs to a specific target, such as a tumor region and control the delivery release, increasing the effectiveness of treatments and reducing possible side effects. Incorporating the enhanced properties of green synthesized nanoparticles loaded into oncology APIs in cancer treatment and diagnosis has opened new possibilities for biomedical applications.

## Figures and Tables

**Table 1:** List of currently developed nanoparticles as drug delivery systems for anticancer application.

Polymeric Nanoparticles	Oncology APIs	Nanoparticle Production	Biological Study	References
Polyhydroxyalkanoate (PHA) nanoparticles	Ellipticine	Emulsification/Solvent evaporation	in vitro	[63,64]
	Cisplatin	Emulsification/Solvent evaporation	in vitro	[65]
	Thymoquinone	Emulsification/Solvent evaporation	in vitro	[66]
	Paclitaxel	Double emulsification/Solvent evaporation	in vitro	[67]
	5-Fluorouracil	Double emulsification/Solvent evaporation	in vitro	[68]
	Etoposide	Solvent evaporation	in vitro	[69]
	Doxorubicin	Double emulsification/Solvent evaporation	in vitro	[70]
	Rhodamine B isothiocyanate (RBITC)	Emulsification/Solvent evaporation	in vitro	[71]
Cyclodextrin (CD) nanoparticles	Docetaxel	Nanoprecipitation	in vitro	[72]
	Camptothecin	Nanoprecipitation	in vitro	[73]
	Acyclovir	Nanoprecipitation	in vitro	[74]
	Paclitaxel	Emulsification/Solvent evaporation method	in vivo	[75]
Poly(thioether-ester) nanoparticles	Zinc phthalocyanine	Thiol-ene miniemulsion polymerization	in vitro	[76]
	Full-spectrum cannabis extract	Thiol-ene miniemulsion and emulsification/Solvent evaporation	in vitro	[77]
	4-nitrochalcone	Thiol-ene miniemulsion polymerization	in vitro	[78]
Poly (lactic co-glycolic acid) (PLGA) nanoparticles	Paclitaxel	Emulsification and nanopracipitation	pre-clinical (mice)	[79]
	Topotecan–tamoxifen	Double emulsification/Solvent evaporation	in vitro	[80]
	Lupeol	Emulsification/Solvent evaporation	in vitro	[81]
	Gemcitabine	Emulsification/Solvent evaporation	in vitro	[82]
	9-nitro-camptothecin	Nanoprecipitation	in vitro	[83]
	Paclitaxel, doxorubicin	Double emulsification/Solvent evaporation	in vitro	[84]
	Paclitaxel	Nanoprecipitation	in vitro	[85]
	Cisplatin	Emulsification/Solvent evaporation	in vitro	[86]
	Paclitaxel/superparamagnetic iron oxide	Emulsification/Solvent evaporation	in vitro	[87]
	Tamoxifen, quercetin	Emulsification/Solvent evaporation	in vitro	[88]
	Docetaxel	Nanoprecipitation	in vitro	[89]
	Δ^9^-Tetrahidrocannabinol	Nanoprecipitation	in vitro	[90]
	Doxorubicin	Solvent displacement	in vitro	[91]
	Paclitaxel	Nanoprecipitation	pre-clinical	[92]
	Bicalutamide	Nanoprecipitation	in vitro	[93]
	siRNA, paclitaxel	Emulsification/Solvent evaporation	in vitro	[94]
	Paclitaxel, doxorubicin	Double emulsification/Solvent evaporation	in vivo	[95]
	Methotrexate	Emulsification and diffusion	in vivo	[96]
	cisplatin	nanoprecipitation	-re-clinical	[97]
Poly (lactic co-glycolic acid) (PLGA) nanoparticles	Doxorubicin	Solvent displacement	in vitro	[98]
	Paclitaxel	Nanoprecipitation	-re-clinical (mice)	[99]
	Curcumin	Nanoprecipitation	in vivo	[100]
	PE38KDL	Double emulsification/Solvent evaporation	pre-clinical (mice)	[101]
	Paclitaxel and magnetic fluid	Emulsification/Solvent evaporation	in vitro	[102]
	Gemcitabine	Double emulsification/Solvent evaporation	in vitro	[103]
	Paclitaxel	Emulsification/Precipitation	in vitro	[104]
	Capecitabine	Emulsification/Solvent evaporation	in vitro	[105]
	SN-38	Emulsification/Solvent evaporation	in vitro	[106]
	BSA	Double emulsification/Solvent evaporation	in vitro	[107]
Chitosan nanoparticles	Quercetin	Coordination reaction	in vitro	[108]
	Curcumin	Ionic gelation method	in vitro	[109]
	Metformin	Ionic gelation method	in vitro and in vivo	[110]
	Chlorin e6	Nonsolvent-aided counterion complexation	in vitro	[111]
	Adriamycin	Dialysis method	in vitro and in vivo	[112]
Polycaprolactone (PCL) nanoparticles	Docetaxel	Emulsification/Solvent evaporation	in vitro	[113]
	Thalidomide	Dialysis method	in vitro and in vivo	[114]
	Docetaxel	Nanoprecipitation technique	in vitro and in vivo	[115]
	Dihydroartemisinin	Self-assembly method	in vitro and in vivo	[116]
	Oxymatrine	pH gradient method	in vitro	[117]
Polycaprolactone (PCL) nanoparticles	Paclitaxel and curcumin	Self-assembly method	in vitro and in vivo	[118]
	Flutamide	Nanoprecipitation method	-	[119]
	5-fluorouracil	Double emulsion technique	in vitro	[120]
	Silibinin	Solvent displacement process	in vitro and in vivo	[121]
Cellulose nanoparticles	Doxorubicin	Self-assembly method	in vitro and in vivo	[122]
	5-Fluorouracil	Co-precipitation method	in vitro	[123]
	coumarin and curcumin	oil in water emulsion technique	in vitro	[124]

**Table 2:** Examples of new oncology APIs for cancer treatment.

Oncology (APIs)	Type of Cancer	Biological Study	References
Quercetin	Breast, lung, liver, colon, intestine	in vitro and in vivo	[134,135,136,137]
Bevacizumab	Colorectal, glibastoma	in vitro and vitro	[138,139,140,141]
Catharanthus roseus extract	Breast, cervical, liver	in vitro	[142,143,144]
Irinotecan	Colorectal, colon, gastric	in vitro	[145,146,147]
Isolated cannabinoids or full-spectrum cannabis extract	Melanoma, glioma, ovarian, leukemia, adenocarcinoma, lung	in vitro, in ovo and in vivo	[77,90,148,149]
Olaparib	Prostate, pancreatic, breast, ovarian	in vitro and vitro	[150,151,152]
Podophyllum extract	Carcinoma, breast	in vitro	[153,154,155]
Temozolomide	Glioma, gliobastoma, lung	in vitro and vitro	[156,157,158]
Vemurafenib	Resistant melanoma	in vitro and vitro	[159,160]
Zinc phthalocyanine	Breast, liver, carcinoma, cervical adenocarcinoma	in vitro and in vivo	[76,161,162]

## Data Availability

Data sharing not applicable.
